# Estrogen-related receptors alpha, beta and gamma expression and function is associated with transcriptional repressor EZH2 in breast carcinoma

**DOI:** 10.1186/s12885-018-4586-0

**Published:** 2018-06-26

**Authors:** Kanchan Kumari, Amit K. Adhya, Arabinda Kumar Rath, P. B. Reddy, Sandip K. Mishra

**Affiliations:** 1 Cancer Biology Laboratory, Department of Gene Function and Regulation, Institute of Life Sciences, Bhubaneswar. Utkal University, Bhubaneswar, Odisha India; 2 Department of Pathology, AIIMS, Bhubaneswar, Odisha India; 3 Hemalata Hospitals, Chandrashekharpur, Bhubaneswar, Odisha India; 4 Department of Microbiology and Biotechnology, Govt. PG College Ratlam, Ratlam, MP India

**Keywords:** EZH2, Orphan nuclear receptors, Breast cancer

## Abstract

**Background:**

Orphan nuclear receptors ERRα, ERRβ and ERRγ that belong to NR3B or type IV nuclear receptor family are well studied for their role in breast cancer pathophysiology. Their homology with the canonical estrogen receptor dictates their possible contributing role in mammary gland development and disease. Although function and regulation of ERRα, ERRγ and less about ERRβ is reported, role of histone methylation in their altered expression in cancer cells is not studied. Transcriptional activity of nuclear receptors depends on co-regulatory proteins. The present study for the first time gives an insight into regulation of estrogen-related receptors by histone methylation specifically through methyltransferase EZH2 in breast cancer.

**Methods:**

Expression of ERRα, ERRβ, ERRγ and EZH2 was assessed by immunohistochemistry in four identical tissue array slides that were prepared as per the protocol. The array slides were stained with ERRα, ERRβ, ERRγ and EZH2 simultaneously. Array data was correlated with expression in MERAV expression dataset. Pearson correlation coeficient r was calculated from the partial matrix expression values available at MERAV database to study the strength of association between EZH2 and three orphan nuclear receptors under study. By western blot and real time PCR, their correlated expression was studied in breast cancer cell lines MCF-7, MDA-MB-231, T47D and MDA-MB-453 including normal breast epithelial MCF-10A cells at both protein and RNA level. Regulation of ERRα, ERRβ, ERRγ by EZH2 was further investigated upon overexpression and silencing of EZH2. The interaction between ERRs and EZH2 was validated in vivo by CHIP-qPCR.

**Results:**

We found a negative correlation between estrogen-related receptors and Enhancer of Zeste Homolog 2, a global repressor gene. Immunohistochemistry in primary breast tumors of different grades showed a correlated expression of estrogen-related receptors and EZH2. Their correlated expression was further validated using online MERAV expression dataset where a negative correlation of variable strengths was observed in breast cancer. Ectopic expression of EZH2 in low EZH2-expressing normal breast epithelial cells abrogated their expression and at the same time, its silencing enhanced the expression of estrogen-related receptors in cancerous cells. Global occupancy of EZH2 on ERRα and ERRβ was observed in-vivo.

**Conclusion:**

Our findings identify EZH2 as a relevant coregulator for estrogen-related receptors in breast carcinoma.

**Electronic supplementary material:**

The online version of this article (10.1186/s12885-018-4586-0) contains supplementary material, which is available to authorized users.

## Background

The second leading cause of the cancer related deaths and the most common cancer evident in females worldwide is breast cancer. Based on the expression of estrogen/progesterone receptor and human epidermal growth receptor 2, there are four major molecular intrinsic subtypes of breast cancer- luminal A (ER+/HER^−^), luminal B (ER+/HER2-or HER2+), triple negative/basal type and HER2 type. Apart from these receptors there are receptors called estrogen-related receptors (ERRs) which share about 68% sequence homology in DNA binding domain (DBD) and significant sequence homology in ligand binding domain (LBD) with estrogen receptor [1]. No natural ligand is found to bind to these receptors giving them their names as orphan nuclear receptors [2]. Three closely related members ERRα, ERRβ and ERRγ constitute the ERR family. Among these, ERRα and ERRγ play significant role as both transcriptional activator as well as repressor [3–11] in cancer and metabolism [1]. Less studied ERRβ [12, 13] expression is lost during cancer progression, which indicates its tumor suppressive role, that still needs to be validated. Although association of ERRs with cancer is evident, fewer studies are there to address their amplified or reduced expression in breast cancer. Upon phosphorylation and PGC-1α mediated regulation of most widely studied ERRα is reported [14, 15]. However, regulation of ERRβ and ERRγ in cancer is completely unknown. Unlike genetic changes, which are reversible, the capricious epigenetic alterations have evolved as captivating curative targets [16–18]. Dramatic increase in the experimental data in the epigenetic area gives the idea of the significance of epigenetic modifications in various stages of tumor progression. Enhancer of zeste homolog 2(EZH2), the catalytic component of polycomb repressive complex 2(PRC2) has been uncovered as an active transcriptional repressor. Amplified EZH2 expression results into deregulation of various genes relevant to signaling pathways in cancer and stem cell biology. A better understanding of regulation of orphan nuclear receptors through various epigenetic modifications might provide various opportunities for developing potential therapeutic targets. Present study investigates the role of EZH2 in regulation of ERRs in breast cancer. A significant association was found between estrogen-related receptors and EZH2. Existing negative correlation between them and recruitment of EZH2 on ERRα and ERRβ confirmed the ongoing in-vivo interaction between them. Overall, our results identify EZH2 as a functional coregulator for estrogen-related receptors especially ERRα and ERRβ in breast carcinoma.

## Methods

### Study approval and ethics statement:

All breast cancer specimens were collected with written informed consents from the patients and were approved by Institutional Human Ethical Committee (Institute of Life Sciences, Bhubaneswar, India). All human tumor samples were handled in accordance with an approved protocol of human ethical committee.

### Human breast Cancer patient samples

Nineteen histologically similar breast tissues both cancerous and non-cancerous tissues (Additional file 1: Table S1) were used to prepare four identical tissue array slides. Using immunohistochemistry, EZH2, ERRα, ERRβ and ERRγ expression was simultaneously assessed in the breast tissue array.

### Immunohistochemistry

Immunohistochemistry in array slides of patient samples was performed as previously described [19]. Slides were incubated with primary antibodies EZH2 (1:100) or ERRα (1:50) or ERRβ (1:50) or ERRγ (1:50) overnight at 4 °C and then subjected to incubation with anti-mouse/rabbit IgG secondary antibody for 1 h. Diaminobenzidine was used to detect the immunoreactivity. Slides were subsequently stained with haematoxylin and processed further. External negative control was taken for non-specific staining by primary antibody (Additional file 1: Figure S3). Stained slides were observed under light microscope (Leica DM500) and images were captured at 4X and 40X magnification. Pathologist scored all the stained slides as previously described [20]. Briefly, the staining intensity of cancerous cells was scored as: absent or weak, 1 point; moderate, 2 points; and strong, 3 points. Percentage positive tumor cells were scored as: 0 for percent of cells < 1, 1 for percent of cells between 1 and 10, 2 for percent of cells between 11 and 33, 3 for percent of cells between 34 and 66 and 4 for percent of cells between 67 and 100. Q score was calculated by multiplying intensity score by the score for percentage of antibody positive cancer cells.

### Cell culture

MCF-7, T47D and MDA-MB-231 breast carcinoma cells were purchased from cell repository of National Centre for Cell Science Pune, Maharashtra, India (Additional file 1: Table S2) and were independently authenticated by STR analysis at Institute of Life Sciences, Bhubaneswar. T47D and MDA-MB-231 cells were maintained in Roswell Park Memorial Institute 1640 medium (RPMI) whereas MCF-7 was maintained in Dulbecco’s Modified Eagle’s Medium (DMEM) containing 10% fetal bovine serum supplemented with penicillin-streptomycin at 37 °C, 5% CO_2_ and 95% humidity. MCF10A, a kind gift from Dr. Annapoorni Rangaranjan (IISC, Bangalore, India) was maintained in DMEM F12 containing horse serum (5%) supplemented with hydrocortisone (0.5 mg/ml), EGF (20 ng/ml), insulin(10 μg/ml), cholera toxin (100 ng/ml) and penicillin-streptomycin at 37 °C, 5% CO_2_ and 95% humidity as previously described [21]. Cells were transfected with pCMV-EZH2 or EZH2si using Lipofectamine 3000 (Invitrogen) according to manufacturer’s protocol. No ethical approval or informed consent was required to use any of the above-mentioned cell lines.

### Online dataset

To investigate the association between EZH2 and estrogen-related receptors, we used online Metabolic gEne RApid Visualizer (MERAV) database (http://merav.wi.mit.edu/). MERAV is designed to analyze normalized human gene expression across 4454 arrays. Our study includes 196 different established and patient derived breast cancer cell line and 332 primary breast tumors of different grades & histology types available in the dataset. The partial matrix provided in the database was used to calculate the Pearson correlation coefficient as a measure of strength of association. Each relative expression value was taken as a single data point.

### Plasmids, cell transfection

Transient knockdown of EZH2 was performed by transfecting 80 pmoles of siRNA clusters (EZH2 antisense sequences: 5′-GGG-AAA-GUG-UAU-GAU-AAA-U55–3′, 5’-AUU-UAU-CAU-ACA-CUU-UCC-C55–3′, 5’-CAC-AAG-UCA-UCC-CAU-UAA-A55–3′, 5’-UUU-AAU-GGG-AUG-ACU-UGU-G55–3′, 5′-GGA-UGG-UAC-UUU-CAU-UGA-A55–3′, 5’-UUC-AAU-GAA-AGU-ACC-AUC-C55–3′) (Eurogentec). Universal negative Control siRNA (Eurogentec) was used as mock. Transient overexpression of EZH2 was performed by transfecting pCMV-EZH2 (Addgene, 24,230) in breast cancer cell lines. Breast cancer cells were transfected using Lipofectamine 3000 (Invitrogen) according to manufacturer’s protocol.

### Western blot assay

Whole cell lysates were prepared using RIPA lysis buffer that consisted of 20 mM Tris-HCl (pH 7.5) 150 mM NaCl, 1 mM Na_2_EDTA, 1 mM EGTA, 1% triton X, 1% sodium deoxycholate, 2.5 mM sodium pyrophosphate, 1 mM β-glycerophosphate, 1 mM Na_3_VO4 and 1 μg/ml protease inhibitor. Equal amount of lysates were electrophoresed on 10% SDS-polyacrylamide gel. The proteins were transferred onto Polyvinylidene difluoride (PVDF) membrane. After blocking the membrane in 5% skimmed milk in tris-buffered saline (TBS) and Polysorbate 20 (Tween 20) TBS-T, incubation was done with primary antibodies against EZH2, ERRα, ERRβ and ERRγ overnight (List of reagents provided in the Additional file 1: Table S2). After washing, the membrane was incubated with anti-rabbit or anti-mouse horseradish peroxidase conjugated secondary antibody for one hour. After secondary antibody wash, the blot was developed for specific proteins using western bright ECL-HRP for X-ray Film Kit in Chemi-Doc (Bio-Rad).

### RNA isolation and quantitative real time PCR

Total RNA was isolated from cells using Trizol (Sigma) as previously described [22]. Equal amount of DNase I treated RNA samples were used to prepare cDNA using SuperScript® First-Strand Synthesis System for RT-PCR (Invitrogen, Carlsbad, CA) as per the manufacturer’s instructions. Quantitative real time PCR was performed on Roche platform using SYBR Green (Thermo scientific) as per the guidelines using gene or site-specific primers (Additional file 1: Table S3). The mRNA level and fold change for each gene compared to control was calculated using value of cycle threshold value taking glyceraldehyde 3-phosphate dehydrogenase for normalization.

### Chromatin immunoprecipitation assay

Breast cancer cells were grown to 90% confluence. CHIP assay was performed with anti-EZH2 as previously described [23] . Briefly, cells were cross-linked with 1% (*v*/v) formaldehyde, lysed in SDS lysis buffer and then sonication was done to obtain 200 bp–500 bp DNA fragments. Keeping aside the input control, the lysate was equally divided for negative control IgG and antibody of interest. De-crosslinking followed Immunoprecipitation with antibody after which DNA was eluted for CHIP-qPCR. The fold enrichment was determined as described previously [24].

### Statistical analyses

Throughout the current study, two-tailed paired Student t-test, One-way ANOVA was performed to test the statistical significance of differences between the experimental groups using the software GraphPad Prism v5.01. Differences in data with values of * *P* < 0.05, ** *P* < 0.005 and *** *P* < 0.001 were considered statistically significant. Pearson correlation coefficient (r) was calculated using the above mentioned software.

## Results

### Expression of ERRα, ERRβ, ERRγ and EZH2 in primary breast tumors of different grades

**Fig. 1:**
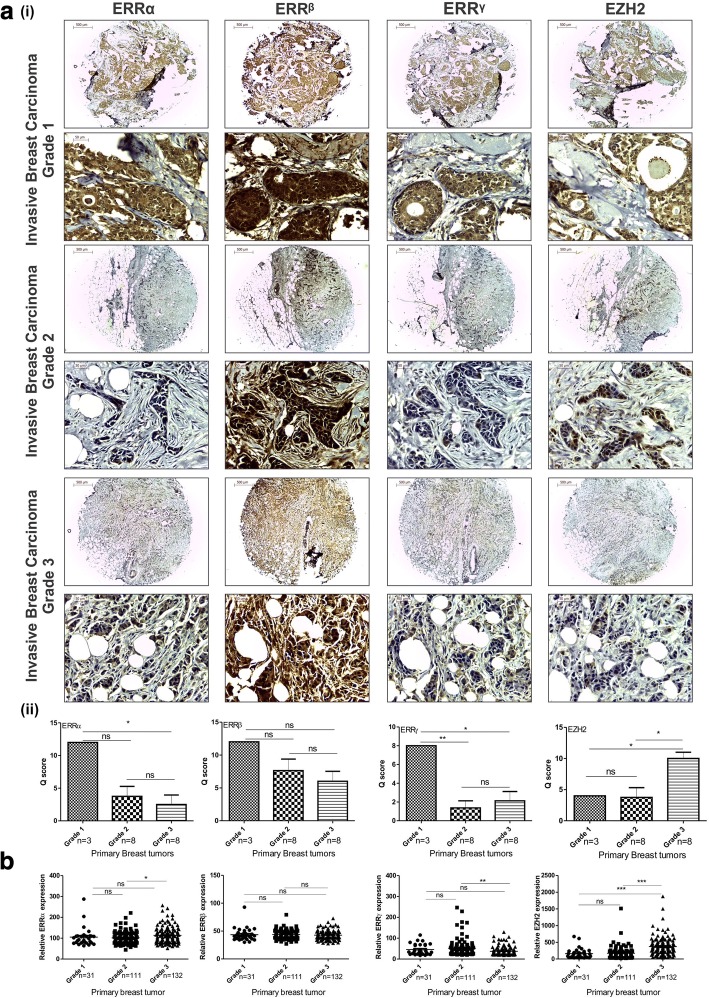
Negatively correlated expression of ERRα, ERRβ & ERRγ and EZH2 in primary breast tumor tissues. **a** (i), Expression of ERRα, ERRβ, ERRγ and EZH2 in three different grades of breast tumors was visualized by immunohistochemistry (Magnification 4X (Scale bar: 500 μm), 40X (Scale bar: 50 μm)). (ii), Graphs show the Q-score for the expression of genes in the breast tissues. **b**, Relative expression of ERRα, ERRβ, ERRγ and EZH2 in different grades of breast tumor as observed in MERAV online expression dataset. One-way ANOVA was used for statistical analysis for experiments done in triplicate.* *P* < 0.05, ** *P* < 0.005, *** *P* < 0.001

To study the expression of EZH2 and ERRs in breast cancer patient samples, immunohistochemistry was performed simultaneously in four identical tissue arrays consisting nineteen cases with less histology variances. Although the expression of ERRα, ERRβ and ERRγ was not found to be tumor grade dependent, notable decrease in the trend of expression of ERRα, ERRβ and ERRγ was detected in increasing grade of breast tumor unlike that of EZH2, which showed a subsequent increased expression (Fig. 1a). Relative expression observed in online MERAV expression dataset showed an enhanced EZH2 expression in primary breast tumors of increasing grade. However, similar to the pattern of expression observed in tissue arrays, the expression of ERRs was observed to be tumor grade independent (Fig. 1b).

### In comparison to normal breast epithelial cells ERRα, ERRβ, ERRγ and EZH2 differentially express in breast cancerous cells

**Fig. 2:**
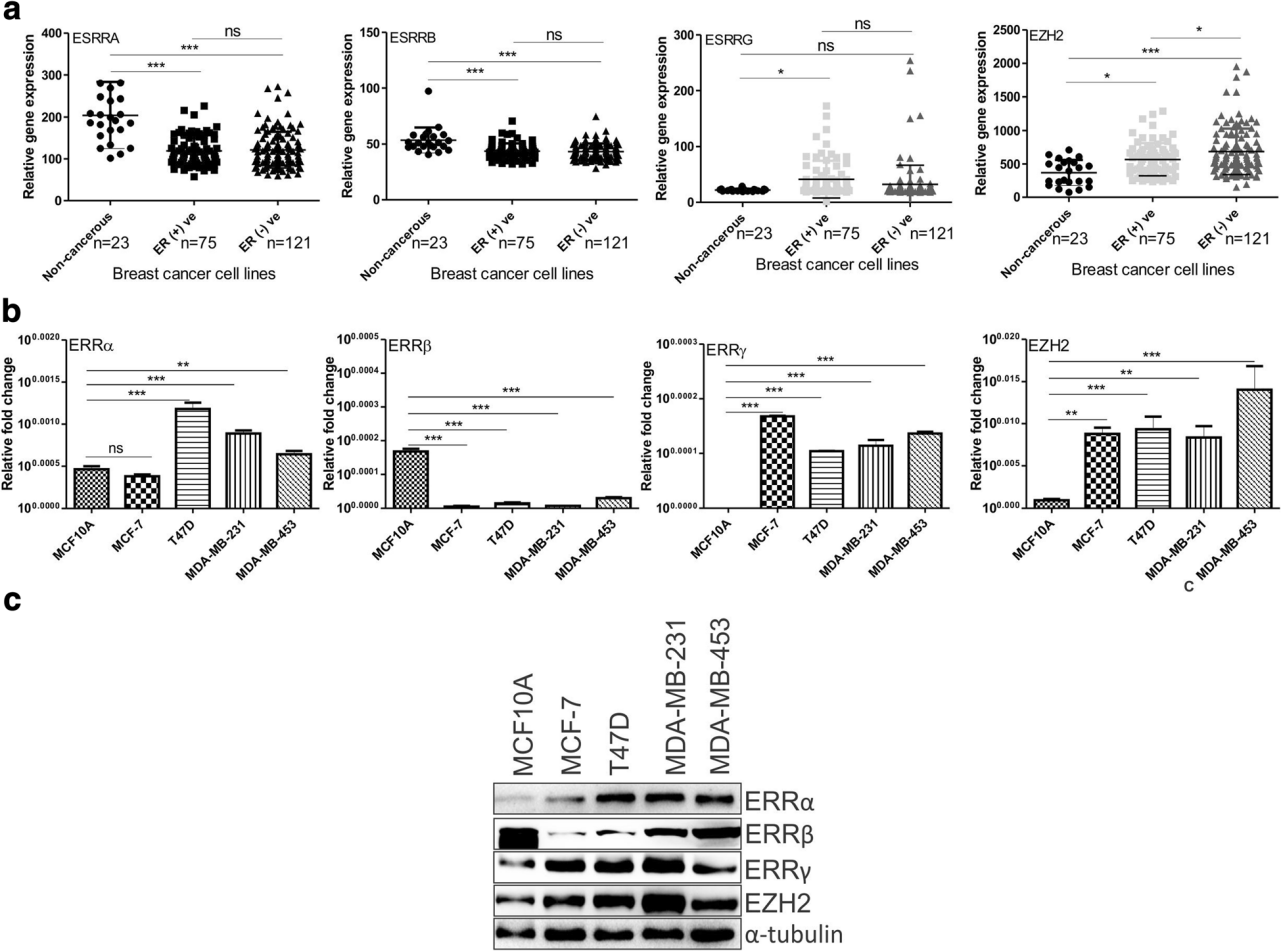
Differential expression of ERRα, ERRβ, ERRγ and EZH2 in normal and cancerous breast cell lines. **a**, Scatter plot shows relative expression of estrogen-related receptors and EZH2 in breast cancerous and non-cancerous cell line dataset of MERAV. **b**, mRNA expression level of orphan nuclear receptors in breast cancer cell lines including normal breast epithelial cells. **c**, Immunoblot shows the protein expression of the genes in different breast cells. Graphs were plotted with SD, which is calculated from three independent experiments. One-way ANOVA was used for statistical analysis for experiments done in triplicate.* *P* < 0.05, ** *P* < 0.005, *** *P* < 0.001

To investigate the expression pattern of EZH2 and orphan nuclear receptors in cell lines, we checked their expression using quantitative real time PCR & immunoblot and by analyzing online data as well. In MERAV breast cancer cell line dataset, expression of ERRα and ERRβ displayed a reduced expression trend in cancerous cells in comparison to non-cancerous breast cells. However, expression of ERRγ showed no specific trend. Reduced ERRγ expression was observed in noncancerous breast cells in comparison to cancerous cells (Fig. 2a). Similar to expression found in primary breast tumors, increased expression of EZH2 was evidenced in breast cancer cells in comparison to normal breast epithelial cells both in MERAV dataset and cells under study in the laboratory. In real time and western blot assay, ERRα expression displayed no specific trend; ERRβ was highly expressed in MCF10A and less expressed in cancerous cells; ERRγ expressed in both ER + ve and ER –ve cancerous cells but its reduced expression was found in normal breast epithelial MCF-10A cells (Fig. 2b, c). The expression pattern of EZH2 and ERRβ cells lines indicated a negative correlation between them, but the expression of ERRα and ERRγ showed not such correlation. The significance of the data lies in the overall trend in the expression of EZH2 and ERRs in various breast cell lines.

### Correlation of estrogen-related receptors alpha beta and gamma with EZH2 expression

**Fig. 3:**
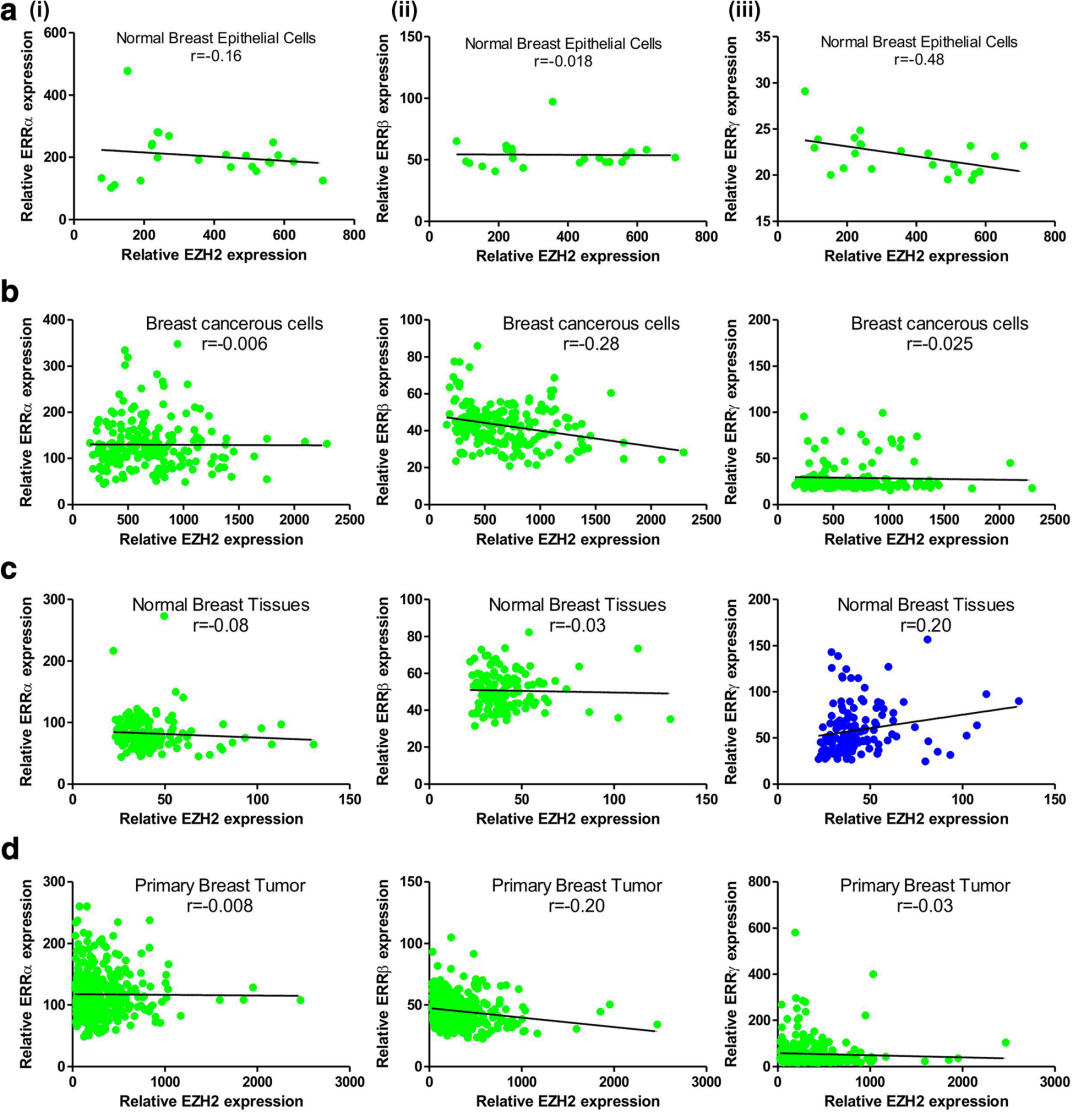
Orphan nuclear receptors shares a negative correlation with EZH2 in breast cancer. Correlation of EZH2 with ERRα(i), ERRβ(ii) and ERRγ(iii) in normal breast epithelial cells (**a**), breast cancerous cells (**b**), normal breast tissues (**c**) and primary breast tumor (**d**) as evidenced in MERAV expression dataset. Graph was plotted and Pearson correlation coefficient was computed using GraphPad Prism software

Correlation is a statistical method that may be used to access the association between two genes. Pearson correlation coefficient is used as a measure of relationship between genes in terms of their expression [25]. A correlation coefficient of zero indicates that no linear relationship exists between two genes, and a correlation coefficient of − 1 or + 1 indicates a perfect linear relationship. The strength of relationship can be anywhere between − 1 and + 1. If the coefficient is a positive number, the variables are positively related. On the other hand, when the coefficient is a negative number, the expression of genes are inversely negatively related. If EZH2 regulates the expression of ERRs by its methyltransferase activity, a negative value of Pearson correlation coefficient is expected. Therefore, to define the type of association between EZH2 and ERRs, Pearson correlation coefficient values were calculated using the partial matrix values provided in the MERAV database. In normal breast epithelial cells, a negative correlation existed between relative expression of all three nuclear receptors and EZH2 (Fig. 3a). However a strong correlation was found between ERRγ (*r* = − 0.48) and EZH2 in comparison to ERRα (*r* = − 0.16) and ERRβ (*r* = − 0.018). A different strength of correlation was observed between ERRs and EZH2 in breast cancerous cells (Fig. 3b). A comparatively strong negative correlation was found between ERRβ and EZH2 (*r* = − 0.28) as between EZH2 and ERRα or ERRγ. In normal breast tissues, although weak, but a negative correlation was observed between EZH2 and ERRα or ERRβ, however a strong positive correlation was found to exist between ERRγ and EZH2 (*r* = 0.2) opposite to what observed in normal breast epithelial cells (Fig. 3c). Similar pattern of correlation as found in cancerous breast cells was evidenced in cancerous breast tissues with strong association between EZH2 and ERRβ (r = − 0.2) (Fig. 3d).

### Ectopic expression of EZH2 reduces the expression of orphan nuclear receptors in normal breast epithelial MCF10A cells

**Fig. 4:**
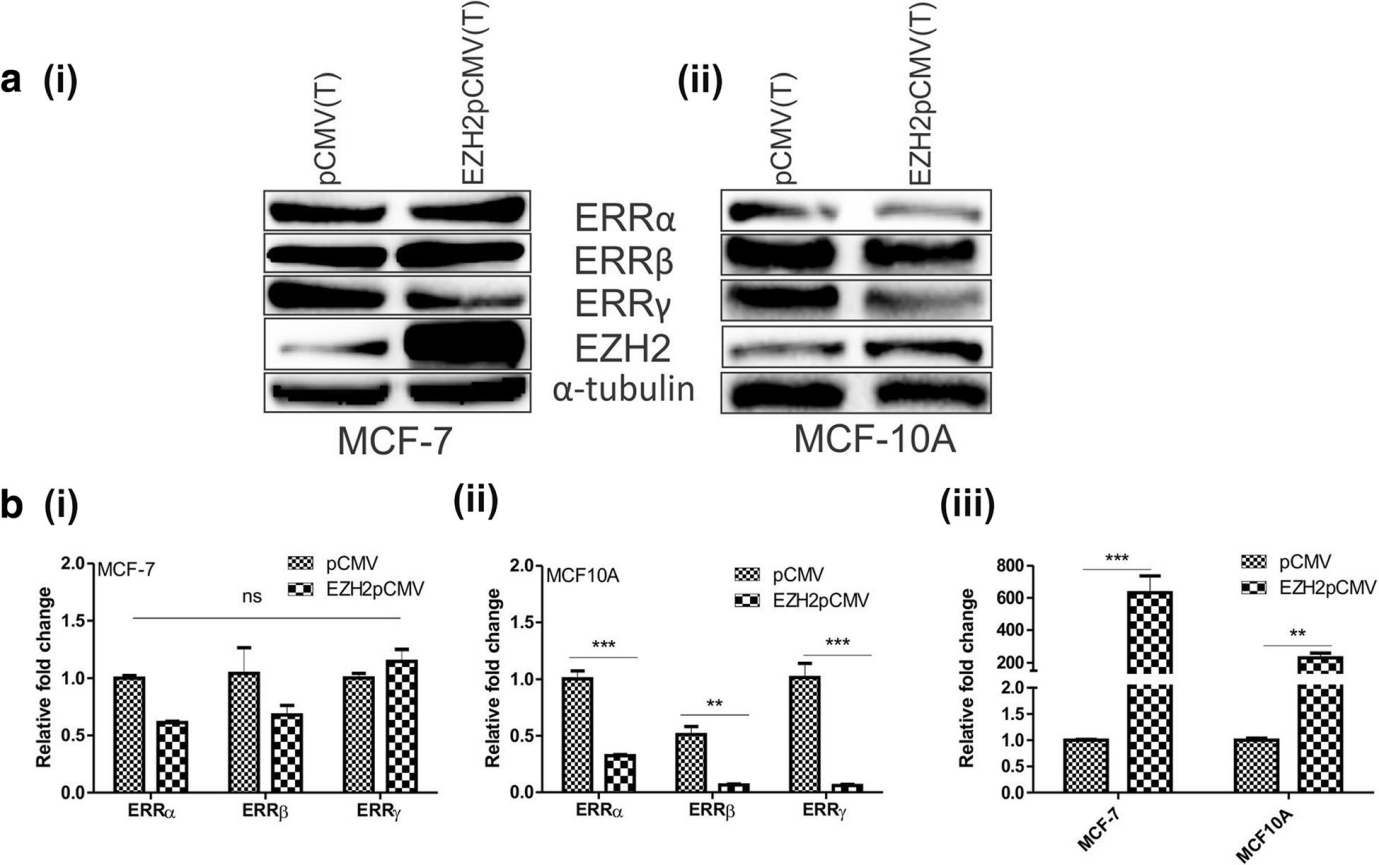
EZH2 overexpression in normal breast epithelial cells affects the expression of orphan nuclear receptors. **a**, Western blot shows the alteration in the expression of ERRs upon ectopic expression of EZH2 in (i), MCF-7 cells and in normal breast epithelial (ii) MCF10A cells. **b**, Transcript level of ERRs in (i) MCF-7 and (ii) MDA-MB-231 upon transfection of cDNA construct of EZH2 (iii). Graphs were plotted with SD, which is calculated from three independent experiments. One-way ANOVA was used for statistical analysis for experiments done in triplicate. ** *P* < 0.005, *** *P* < 0.001

Further, to understand the regulation of orphan nuclear receptors by EZH2, we first checked their expression level after over-expressing EZH2 in cancerous MCF-7 cells and non-cancerous MCF10A cells (Fig. 4b (iii)). In high EZH2 expressing MCF-7 breast cancerous cells, the effect of EZH2 overexpression was not significant at both protein (Fig. 4a (i)) and RNA level (Fig. 4b (i)). However in less EZH2 harboring normal breast epithelial MCF10A cells, EZH2 overexpression resulted into significant reduced level of ERRα, ERRβ and ERRγ at protein (Fig. 4a (ii)) as well as RNA level (Fig. 4b (ii)).

### Silencing of EZH2 increases the expression of orphan nuclear receptors

**Fig. 5:**
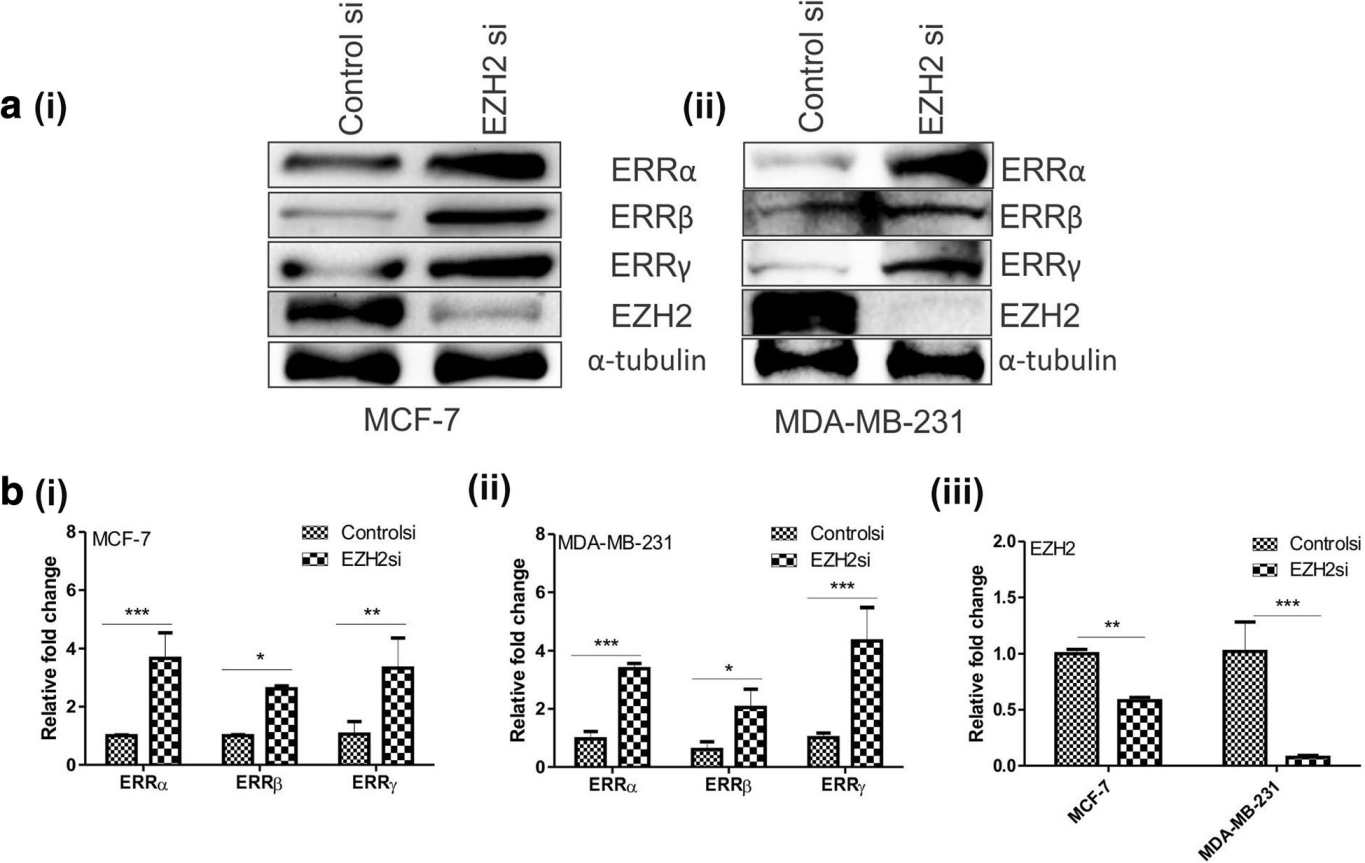
Silencing of EZH2 enhances the expression of ERRα, ERRβ, ERRγ and EZH2 in breast cancerous cell lines. **a**, Western blot shows the enriched expression of ERRs upon EZH2 silencing in (i), MCF-7 and (ii) MDA-MB-231 cancerous cells. **b**, Graphs show the transcript level of ERRs in (i) MCF-7 and (ii) MDA-MB-231 cells upon transfection of EZH2si (iii). Graphs were plotted with SD, which is calculated from three independent experiments. One-way ANOVA was used for statistical analysis for experiments done in triplicate.* *P* < 0.05, ** *P* < 0.005, *** *P* < 0.001

To further validate the EZH2-mediated regulation of ERRs, we next checked their expression upon EZH2 silencing. Transfection of EZH2si in breast cancer cells considerably reduced its level (Fig. 5b (iii)). In both estrogen receptor positive MCF-7 and estrogen receptor negative MDA-MB-231 breast cancer cells, significantly increased protein as well as RNA expression of ERRα, ERRβ and ERRγ was detected (Fig. 5a and b).

### EZH2 regulates ERRα and ERRβ by direct binding

**Fig. 6:**
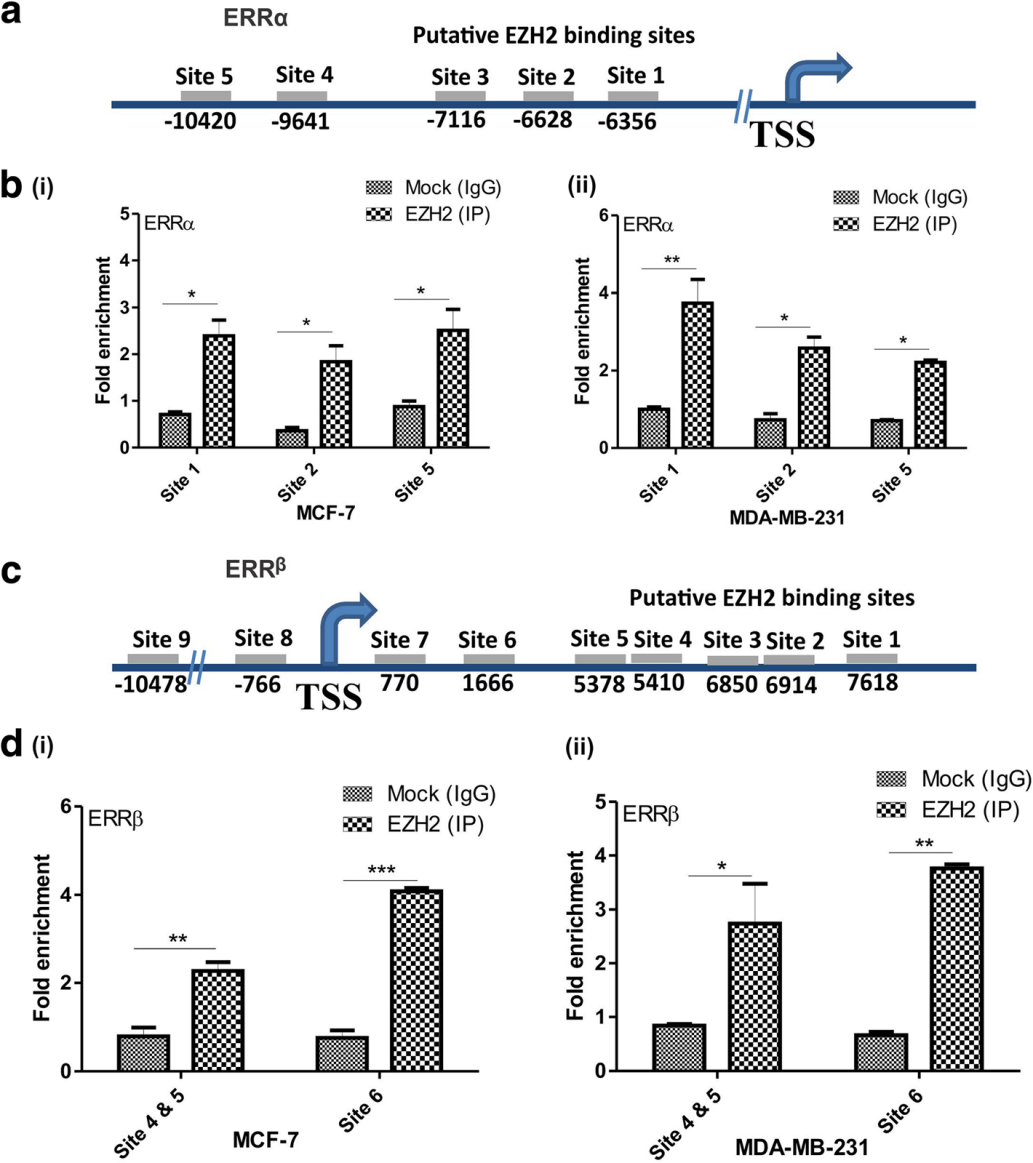
EZH2 interacts with ERRα and ERRβ in-vivo. **a**, Diagram shows the putative EZH2 binding sites on ERRα promoter. **b**, Graphs show the fold enrichment of EZH2 at two binding sites at ERRα promoter in MCF-7(i) and MDA-MB-231(ii) cells. **c**. Diagram shows the putative EZH2 binding sites on ERRβ promoter and downstream genomic loci. D, Graphs show the fold enrichment of EZH2 at two binding sites at in-gene region of ERRβ in MCF-7(i) and MDA-MB-231(ii) cells

Further, to confirm the interaction of orphan nuclear receptors and polycomb group protein EZH2 in-vivo, we explored the CHIP-seq data performed in epithelial ovarian cancer cells upon EZH2 knockdown [26]. In the ChIP-seq data, EZH2 was found to directly bind to the genomic loci of ERRα at five putative binding sites (Fig. 6a) and ERRβ at 21 putative binding sites (we explored upto 10 Kb upstream and downstream of TSS) (Fig. 6c). The data suggested the binding of EZH2 on promoter region of ERRα and at both promoter as well as in-gene region of ERRβ for regulation. To confirm the protein-DNA binding we performed CHIP-qPCR by immunoprecipitation with EZH2 in MCF-7 breast cancer cells. Primers specific to the suggested region of binding were used for amplification. Input control was used for the amount of chromatin considered for the study and IgG was taken as negative control. By CHIP-qPCR three sites of ERRα (Fig. 6b) and two sites on ERRβ (Fig. 6d) was found to be occupied by EZH2 in both MCF-7 and MDA-MB-231 breast cancer cells (Additional file 1: Figure S1 and S2 shows the agarose gel images of the CHIP –qPCR product). However, as no EZH2 binding sites were observed on ERRγ, regulation of ERRγ by EZH2 may not be direct.

## Discussion

Significant homology with estrogen receptors dictates the role of estrogen-related receptors (ERRs) in disease progression. Association of estrogen receptors with epigenetics [27, 28] reflects the possible involvement of epigenetics in the regulation of estrogen-related receptors as well. Reduced expression of ERRβ in breast cancer cells also anticipates the participation of repressor proteins for its regulation. Inter and intra-tumoral heterogeneity underlies the diverse pattern of expression of estrogen-related receptors in different grades of tumor as displayed by breast tumor dataset of MERAV. The MERAV breast cancer expression dataset consists of different histological types of tumor such as IDC, Ductal, Papillary, Medullary, Lobular, Inflammatory, Mucinous, Metaplastic Squamous Carcinoma etc. In addition, varied expression pattern of estrogen-related receptors observed in breast cells and tissues can be explained from the point that cell lines are derived from tumors and are grown and sub-cultured in vitro. Cell lines acquire changes in the process of immortalization and maintenance in culture. Here, in this study, we have shown the expression of ERRs in five different breast cell lines and histologically similar primary breast tumors; however, the online dataset displays the pattern observed in large number of cell lines maintained in different laboratories under different conditions, which answers the difference in the expression pattern [29–31]. A varied strength of negative correlation between EZH2 and ERRs further indicated the possible interaction between them. To investigate their association with global histone methyltransferase EZH2 we first checked the alteration in expression of ERRs upon EZH2 overexpression in cancerous ER positive MCF-7 cells, where insignificant or no change in expression of ERRs was found. In cancerous breast cells, endogenous level of EZH2 is high, thus upon its over-expression, it may reach its saturated level of expression. As evident from the graph of Fig. 4b(i), although the effect of EZH2 overexpression shown non-significant effect on the expression of ERRs, when closely analyzed the percent knockdown in their expression, about 40% reduction in ERRα and about 35% reduction in ERRβ level was observed. Expression of ERRγ was enhanced by 14%. This suggests that although the influence is not very effective, its effect is considerable for ERRα and ERRβ. The degree of overexpression relative to the native level should vary strongly among the analyzed proteins. The absolute overexpression experiments analyze the consequences of comparably strong production of target proteins independently of their endogenous expression levels [32]. Such as if the endogenous expression of a target protein is 100 molecules/cell, the degree of overexpression will be likely 50,000-fold. If the endogenous expression of a target protein is 100,000 molecules/cell, the overexpression degree will be 50-fold. The fold change in EZH2 mRNA level upon overexpression was about 600 fold, but EZH2 protein was found to be only four fold increased when analyzed by ImageJ quantitation.

However, in low EZH2 expressing normal breast epithelial MCF-10A cells, significant reduced expression of ERRs was evident upon EZH2 overexpression. Significant enhanced expression of ERRα, ERRβ and ERRγ upon EZH2 silencing further strengthened the existing association between them. Occupancy of EZH2 on ERRα and ERRβ in EZH2-CHIP-seq data confirmed the possible interaction between EZH2 and ERRs. Well-studied ERRα and ERRγ are biomarkers in breast cancer [10], but their role and regulation is not clear as they act as both transcriptional activator and repressor [11]. Association with co-activators and co-repressors might be the answers to their differential expression and thus function in cancer. As in CHIP-seq data, EZH2 was not found to occupy ERRγ gene; the positive correlation between them in normal breast tissues supported the existence of an indirect association. Fold enrichment of EZH2 on ERRα and ERRβ in CHIP-qPCR clearly showed in-vivo interaction between them. Although ERRα, ERRβ and ERRγ are prognostic markers for various cancers, their role and regulation is far from being clearly understood. Different study report inconsistent functions of ERRα [11, 33], ERRβ [12, 34, 35] and ERRγ [8, 36]. Association of estrogen-related receptors with coregulators such as AIB1 [37] and EZH2 designates their regulation to be controlled by various factors. A better understanding of the regulation of ERRs will provide new insights into cancer biology.

## Conclusions

Taken together our data suggests the regulation of estrogen-related receptors by global repressor gene, enhancer of zeste homolog 2. Transcription regulation of ERRs by coregulators such as EZH2 supports their inconsistent expression and function that is yet to be defined. Elucidation of such epigenetic regulations of orphan nuclear receptors will be helpful in understanding their role and regulation in breast carcinoma.

## Additional file

Additional file 1: Tissue microarray patient sample details. Table provides the details of reagents used in the study. Table provides the sequence of primers used in the study. Agarose gel picture of CHIP-qPCR product for positive binding sites of EZH2 on ERRα and ERRβ. Agarose gel picture of CHIP-qPCR product for negative binding sites of EZH2 on ERRα and ERRβ Immunohistochemistry negative control cases for primary antibodies ERRα, ERRβ, ERRγ and EZH2. **Table S1.** Tissue microarray patient sample details. Nineteen breast carcinoma patient samples were included in the the study. **Table S2.** Table provides the details of reagents used in the study. All reagents used in the study were obtained from authentic companies. **Table S3.** Table provides the sequence of primers used in the study. **Figure S1.** Agarose gel picture of CHIP-qPCR product for positive binding sites of EZH2 on ERRα and ERRβ. **Figure S2.** Agarose gel picture of CHIP-qPCR product for negative binding sites of EZH2 on ERRα and ERRβ. **Figure S3.** Immunohistochemistry negative control cases for primary antibodies ERRα, ERRβ, ERRγ and EZH2. (PDF 757 kb)

## Abbreviations

AIB1: Amplified in breast cancer-1
EGF: Epidermal growth factor
ERRs: Estrogen-related receptors
EZH2: Enhancer of zeste homolog 2
MERAV: Metabolic gEne RApid Visualizer
pCMV: plasmid cytomegalovirus
r: Pearson correlation coefficient
SD: Standard deviation
STR: Short Tandem Repeat
TSS: Transcription start site
