# Supplementary material for: Estrogen-related receptors alpha, beta and gamma expression and function is associated with transcriptional repressor EZH2 in breast carcinoma
Source: BMC Cancer. 2018 Jun 26;18:690. doi: 10.1186/s12885-018-4586-0 (PMC6019302; doi:10.1186/s12885-018-4586-0)
Supplement: Supplementary file 1 — Tissue microarray patient sample details. Table provides the details of reagents used in the study. Table provides the sequence of primers used in the study. Agarose gel picture of CHIP-qPCR product for positive binding sites of EZH2 on ERRα and ERRβ. Agarose gel picture of CHIP-qPCR product for negative binding sites of EZH2 on ERRα and ERRβ Immunohistochemistry negative control cases for primary antibodies ERRα, ERRβ, ERRγ and EZH2. Table S1. Tissue microarray patient sample details. Nineteen breast carcinoma patient samples were included in the the study. Table S2. Table provides the details of reagents used in the study. All reagents used in the study were obtained from authentic companies. Table S3. Table provides the sequence of primers used in the study. Figure S1. Agarose gel picture of CHIP-qPCR product for positive binding sites of EZH2 on ERRα and ERRβ. Figure S2. Agarose gel picture of CHIP-qPCR product for negative binding sites of EZH2 on ERRα and ERRβ. Figure S3. Immunohistochemistry negative control cases for primary antibodies ERRα, ERRβ, ERRγ and EZH2. (PDF 757 kb) [file 12885_2018_4586_MOESM1_ESM.pdf]

Primary breast tumor details		
S.No.	Breast cancer type	Grade
1	Invasive breast Carcinoma	2
2	Invasive Breast carcinoma	3
3	Invasive Breast Carcinoma	1
4	Invasive Breast Carcinoma	2
5	Invasive Breast carcinoma	3
6	Ductal Carcinoma In Situ	3
7	Invasive Breast Carcinoma	1
8	Invasive lobular carcinoma	3
9	Invasive Breast carcinoma	2
10	Invasive Breast carcinoma	3
11	Invasive Breast carcinoma	3
12	Invasive Breast carcinoma	3
13	Ductal Carcinoma In Situ	2
14	Residual Invasive Breast Carcinoma	2
15	Invasive Breast carcinoma	2
16	Invasive Breast Carcinoma	2
17	Residual Invasive Breast Carcinoma	3
18	Mucinous Carcinoma	1
19	Invasive Breast carcinoma	2

Table S1. **Tissue microarray patient sample details.** Nineteen breast carcinoma patient samples were included in the the study.

Antibody/Reagent/Kit	Catalog no.	Company
α-tubulin antibody	T5168	Sigma Aldrich, USA
anti-EZH2 antibody	D2C9-5246	Cell Signaling Technology
anti-Rabbit IgG (whole molecule)–Peroxidase antibody produced in goat secondary antibody	A0545	Sigma Aldrich, USA
anti-mouse HRP conjugated secondary antibody	A3682-1ML	Sigma Aldrich, USA
Anti-ERRα antibody	sc-65715	Santacruz Biotechnology
Anti-ERRβ antibody	sc-376449	Santacruz Biotechnology
Anti-ERRγ antibody	sc-393969	Santacruz Biotechnology
SYBER Green	RT-SY2X-03+NRWOU	Eurogentech
SuperScript® First-Strand Synthesis System for RT-PCR	11904018	Invitrogen, Carlsbad, CA
Dnase I Kit	AMPD1-1KT	Sigma Aldrich, USA
Lipofectamine 3000		Invitrogen
PVDF membrane	88518, 0.45μm,	Thermo scientific
Skimmed milk	RM1254-500G	Himedia
ECL-HRP for X-ray Film Kit	K-12045-D50	
Trizol	T9424	Sigma Aldrich, USA
MCF-7 cell line	ATCC_HTB-22™	NCCS Pune, Maharastra,India
T47D cell line	ATCC_HTB-133™	NCCS Pune, Maharastra,India
MDA-MB-231 cell line	ATCC_HTB-26™	NCCS Pune, Maharastra,India

Table S2. Table provides the details of reagents/cell lines used in the study. All reagents used in the study were obtained from authentic companies.

**Primer name**	**Sequence (5'->3')**
ESRRA SITE 1 F	GTCCCAAATATTGCACGAGATA
ESRRA SITE 1 R	ATCAAACCCCAACCCTCTTTT
ESRRA SITE 2 F	GAAACCCCGTCTCTACTAAAA
ESRRA SITE 2 R	TATCTCGTGCAATATTTGGGAC
ESRRA SITE 3 F	GAAACCCCGTCTCTACTAAAA
ESRRA SITE 3 R	TATCTCGTGCAATATTTGGGAC
ESRRA SITE 4 F	GACTAAGTCTTGCTAATGAGGA
ESRRA SITE 4 R	GACTATTTTGAGAGTGCTTGC
ESRRA SITE 5 F	TAGGTGCTGTAGTGTTATATGC
ESRRA SITE 5 R	GTATTTCAATCACCAGGTCATC
ESRRB SITE 1 F	CTTTGGTTTGGTAGAAGGATAGTAG
ESRRB SITE 1 R	CTATGTGTTAGAAGCAAAAGGG
ESRRB SITE 2&3 F	TTGTGGAGCATTTCCTTACA
ESRRB SITE 2&3 R	AAAGGGTTGGGGATCTAAAC
ESRRB SITE 4&5 F	GTTTCTTTCTAGGGCATAAACC
ESRRB SITE 4&5 R	GTTCGATTTTCTCTCTCTTGC
ESRRB SITE 6 F	AGTAGAAATTCAGTGACCACAG
ESRRB SITE 6 R	GGACAAAAGCTATTAGATGCAG
ESRRB SITE 7 F	AAGAGAGGGAAGATACTACCAA
ESRRB SITE 7 R	TCAGAGATCTAGACAAACACAG
ESRRB SITE 8 F	CCAAAGTCACAAGAAACTGAAC
ESRRB SITE 8 R	AGAGAAAGAATACCATACCTCG
ESRRB SITE 9 F	CTAGCACTGAATGTAACAAGAC
ESRRB SITE 9 R	CCTGAATATTGTCTCATGGATC
5’Ex1Ezh2	AGAAGGGACCAGTTTGTTGG
3’Ex1Ezh2	TTCATCAGCTCGTCTGAACC
GAPDH RT FW	AAGATCATCAGCAATGCCTC
GAPDH RT RV	CTCTTCCTCTTGTGCTCTTG
5’Ex6ERRβF	AAGCCATTGACCAAGATTGT
3’EX7ERRβR	GGTCACAGAGAGTGGTCAGG
5’Ex7ERRαF	CTCAGGGGGTAGGAGAGCAC
3’EX7ERRαR	CTCCCTGGCCAAACCCAAAA
5’Ex2ERRγF	CAGAATGTCAAACAAAGATCGACAC
3’EX3ERRγR	GGTTGAACTGTAGCTCCCACTG

Table S3. Table provides the sequence of primers used in the study.

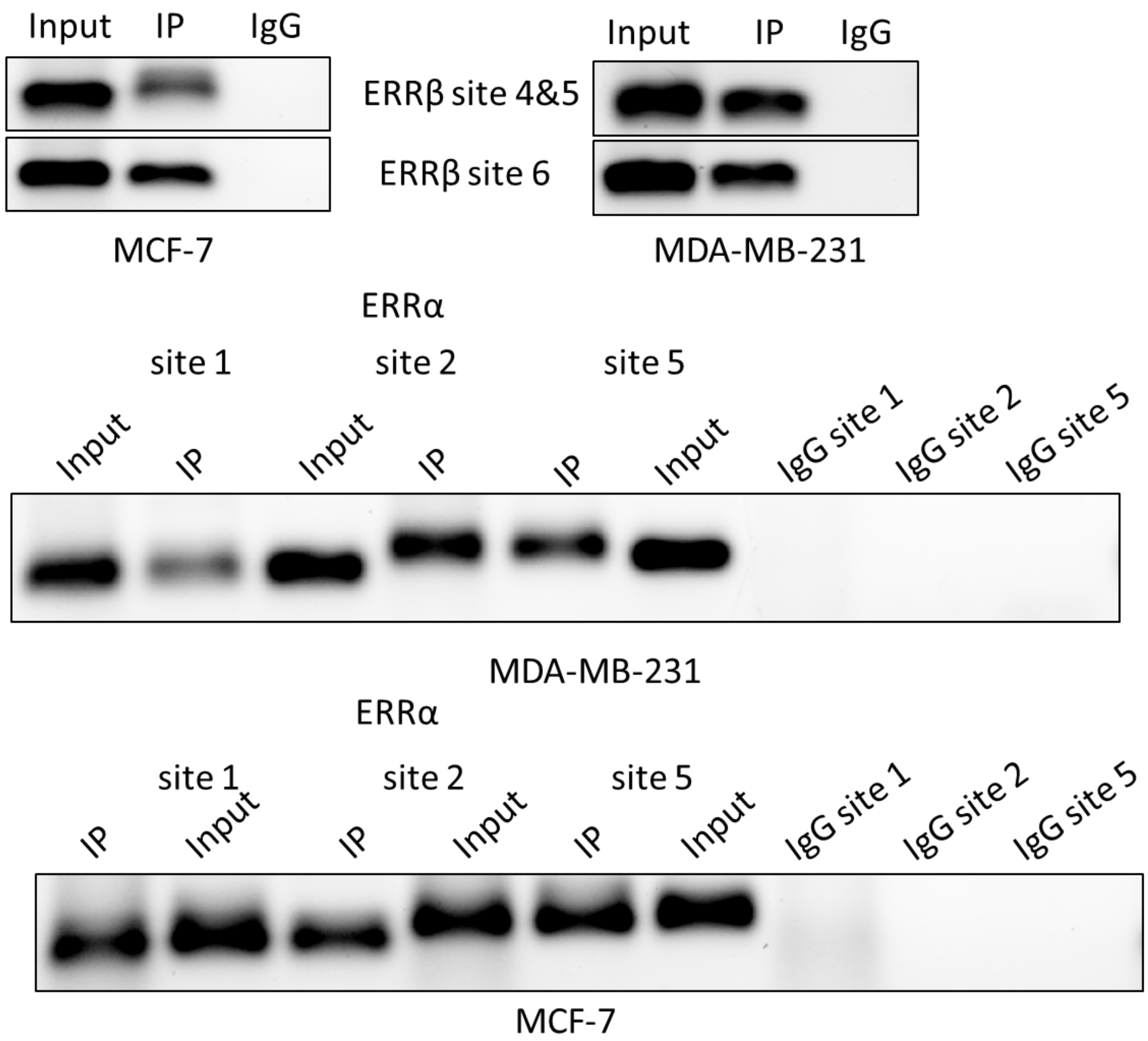

Figure S1: Agarose gel picture of CHIP-qPCR product for positive binding sites of EZH2 on ERRα and ERRβ

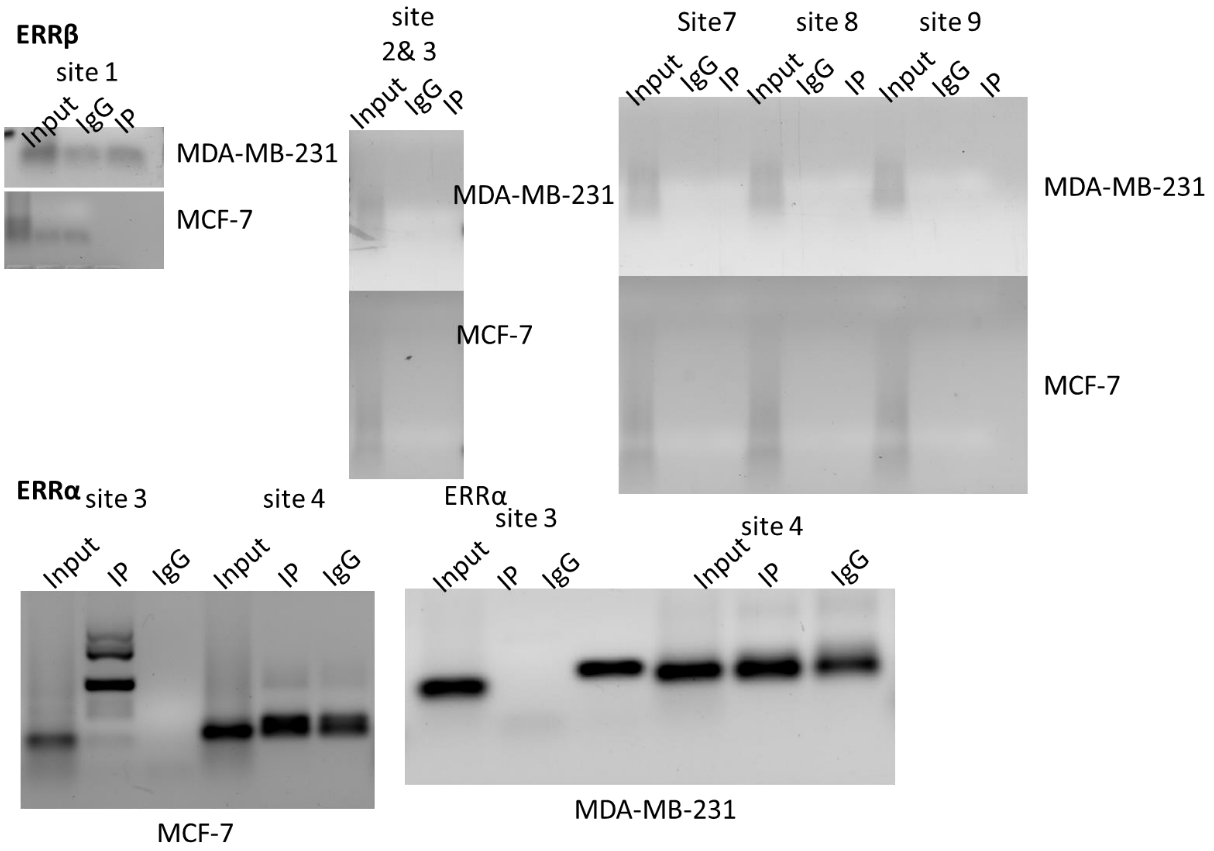

Figure S2: Agarose gel picture of CHIP-qPCR product for negative binding sites of EZH2 on ERRα and ERRβ

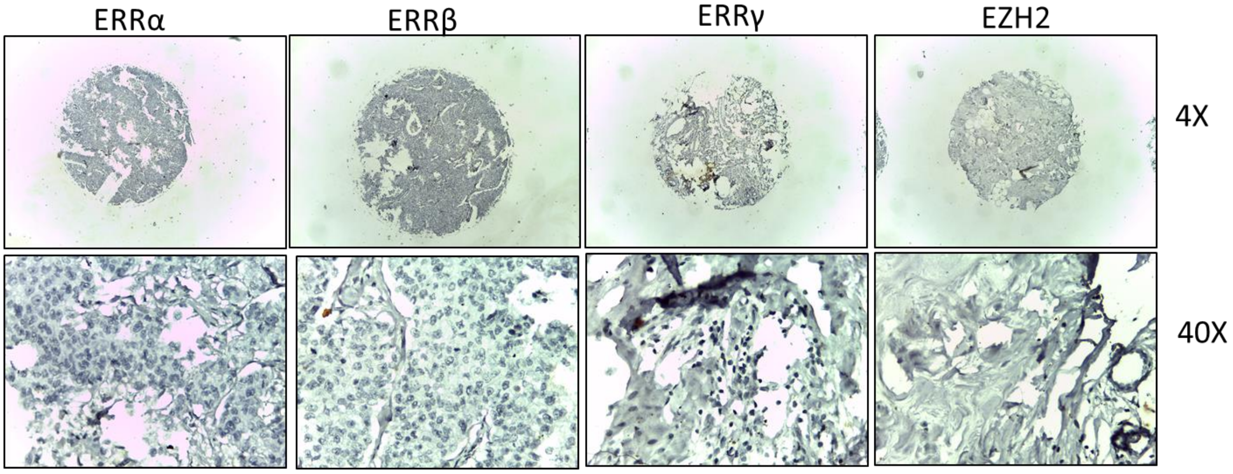

Figure S3: Immunohistochemistry negative control cases for primary antibodies ERRα, ERRβ, ERRγ and EZH2.
